# Vaginal tolerance of CT based image-guided high-dose rate interstitial brachytherapy for gynecological malignancies

**DOI:** 10.1186/1748-717X-9-31

**Published:** 2014-01-23

**Authors:** Naoya Murakami, Takahiro Kasamatsu, Minako Sumi, Ryoichi Yoshimura, Ken Harada, Mayuka Kitaguchi, Shuhei Sekii, Kana Takahashi, Kotaro Yoshio, Koji Inaba, Madoka Morota, Yoshinori Ito, Jun Itami

**Affiliations:** 1 Department of Radiation Oncology, National Cancer Center Hospital, 5-1-1, Tsukiji Chuo-ku, Tokyo 104-0045, Japan; 2 Department of Gynecologic Oncology, National Cancer Center Hospital, 5-1-1, Tsukiji Chuo-ku, Tokyo 104-0045, Japan; 3 Department of Radiology, Showa University School of Medicine, 1-5-8, Hatanodai Shinagawa-ku, Tokyo 142-8666, Japan

**Keywords:** Gynecologic brachytherapy, High-dose-rate brachytherapy, Interstitial brachytherapy, Vaginal ulcer

## Abstract

**Background:**

Purpose of this study was to identify predictors of vaginal ulcer after CT based three-dimensional image-guided high-dose-rate interstitial brachytherapy (HDR-ISBT) for gynecologic malignancies.

**Methods:**

Records were reviewed for 44 female (14 with primary disease and 30 with recurrence) with gynecological malignancies treated with HDR-ISBT with or without external beam radiation therapy. The HDR-ISBT applicator insertion was performed with image guidance by trans-rectal ultrasound and CT.

**Results:**

The median clinical target volume was 35.5 ml (2.4-142.1 ml) and the median delivered dose in equivalent dose in 2 Gy fractions (EQD_2_) for target volume D_90_ was 67.7 Gy (48.8-94.2 Gy, doses of external-beam radiation therapy and brachytherapy were combined). For re-irradiation patients, median EQD_2_ of D_2cc_ for rectum and bladder, D_0.5cc_, D_1cc_, D_2cc_, D_4cc_, D_6cc_ and D_8cc_ for vaginal wall was 91.1 Gy, 100.9 Gy, 260.3 Gy, 212.3 Gy, 170.1 Gy, 117.1 Gy, 105.2 Gy, and 94.7 Gy, respectively. For those without prior radiation therapy, median EQD_2_ of D_2cc_ for rectum and bladder, D_0.5cc_, D_1cc_, D_2cc_, D_4cc_, D_6cc_ and D_8cc_ for vaginal wall was 56.3 Gy, 54.3 Gy, 147.4 Gy, 126.2 Gy, 108.0 Gy, 103.5 Gy, 94.7 Gy, and 80.7 Gy, respectively. Among five patients with vaginal ulcer, three had prior pelvic radiation therapy in their initial treatment and three consequently suffered from fistula formation. On univariate analysis, re-irradiation and vaginal wall D_2cc_ in EQD_2_ was the clinical predictors of vaginal ulcer (*p* = 0.035 and *p* = 0.025, respectively). The ROC analysis revealed that vaginal wall D_2cc_ is the best predictor of vaginal ulcer. The 2-year incidence rates of vaginal ulcer in the patients with vaginal wall D_2cc_ in EQD_2_ equal to or less than 145 Gy and over 145 Gy were 3.7% and 23.5%, respectively, with a statistically significant difference (*p* = 0.026).

**Conclusions:**

Re-irradiation and vaginal D_2cc_ is a significant predictor of vaginal ulcer after HDR-ISBT for gynecologic malignancies. Three-dimensional image-guided treatment planning should be performed to ensure adequate target coverage while minimizing vaginal D_2cc_ in order to avoid vagina ulcer.

## Introduction

High-dose rate intracavitary brachytherapy (HDR-ICBT) is an established method in the management of gynecological malignancies, especially in cervical cancer. However, in patients with a narrow vagina, short uterine cavity, distal vaginal extension, and bulky tumors in which the optimal dose distribution cannot be obtained by intracavitary brachytherapy (ICBT), interstitial brachytherapy (ISBT) is employed. Also in patients with bulky postoperative central pelvic recurrence, ISBT has proven to be effective [1-5]. With the advent of image-guided brachytherapy it has become possible to assess the dose volume histogram (DVH) in brachytherapy. Several studies have validated the D_2cc_ as a predictor of rectal and bladder toxicities for ICBT [6] or for ISBT [7]. D_2cc_ of the rectum and bladder have been introduced into daily clinical practice of gynecological image-guided brachytherapy. However in ICRU 38 vagina was not recognized as organ at risk during brachytherapy tough it is adjacent to target volume and radioactive sources [8].

The purpose of this study was to retrospectively analyze the incidence of vaginal morbidities after HDR-ISBT for gynecological cancers and to find clinical and dosimetric factors which affect the incidence of the vaginal morbidities.

## Methods

The inclusion criteria of this single institutional retrospective study were patients with gynecological malignancies who were treated by HDR-ISBT with or without external beam radiation therapy (EBRT) with a follow-up length exceeding 6 months or more. Patients with distant metastasis outside of pelvis were excluded from current study. HDR-ISBT was applied for both primary and salvage intents. Patients with superficial vaginal disease with thickness less than 5 mm were treated with HDR-ICBT and did not treated by HDR-ISBT; therefore these patients were not included in this analysis. Also HDR-ISBT was not applied for those patients who had distant metastasis or for those patients with far advanced tumors which had not responded to EBRT performed before HDR-ISBT. These patients were treated with EBRT alone. One patient who succumbed to progressive cancer in 5.5 months after ISBT was also excluded in this analysis. The medical records of all patients with gynecological malignancies treated with HDR-ISBT at the National Cancer Center Hospital, Tokyo, Japan, between 2008 and 2011 were retrieved and 44 patients were included in this study.

In the patients without prior pelvic irradiation, pelvic EBRT was delivered before HDR-ISBT. The common EBRT portals were whole pelvic irradiation including gross tumor volume (GTV) with adequate margin as well as the pelvic lymph nodes basin up to the level of the common iliac (L4/5 junction). If the tumor involved the lower third of the vagina, or there were clinically palpable inguinal nodes, inguinal regions were also included in the EBRT portals. The initial 20-40 Gy was delivered to the whole pelvis with a 4-fields box technique and then pelvic irradiation was administered with a central shield being employed to reduce exposure of organs at risk (OAR). The total dose delivered to the pelvic side wall was up to 50 Gy in a conventional fractionation. In patients with a history of prior pelvic radiation therapy or in feeble elderly patients, no EBRT or smaller EBRT fields with a reduced total dose were employed. HDR-ISBT was basically performed after the central shield was inserted. However for those patients treated without EBRT, HDR-ISBT was applied as solitary radiotherapy modality. The detailed procedure of gynecological HDR-ISBT was described elsewhere [9]. In brief, transperineal needle applicator insertion was performed under either general or local anesthesia with the patients in lithotomy position and guided by trans-rectal ultrasound (TRUS) or CT which can be taken with the patients lying in lithotomy position with the applicators in place. For advanced large disease, a Syed-Neblett perineal template (Best Medical International, Inc., Springfield, VA) was used in order to sufficiently cover lateral disease extent. For rather localized small disease, with limited parametrial and/or paracolpial invasion, free-hand needle applicator insertion with or without a vaginal applicator was used with fewer needles inserted compared to the Syed-Neblett perineal template. Treatment planning was performed with brachytherapy planning system (Oncentra^®^ Nucletron, Veenendaal, The Netherlands) using CT images taken by the large bore CT simulator (Aquilion LG^®^, Toshiba, Tokyo, Japan), which allows imaging of the patients in lithotomy position. Although different applicator was used throughout the patients, the calculation method applied was the same. The clinical target volume (CTV) was defined based on the CT image obtained after needle insertion, as well as physical examination immediately before needle insertion, the intra-operative TRUS image and the most recent MRI were also taken into account. Reference points were set on the surface of CTV and prescribed dose was delivered to those points. HDR-ISRT treatment plan was calculated initially by geometrical optimization or volume optimization and then manual graphical modification was followed to enclose the CTV by the prescription dose while minimizing high dose to OAR. The median HDR-ISBT dose was 24 Gy (range, 18-54 Gy), and median HDR-ISBT dose per fraction was 6 Gy (range, 4-6 Gy). HDR-ISBT was performed twice daily with each fraction 6 hours apart. HDR-ISBT was performed with MicroSelectron HDR (Nucletron, Veenendaal, The Netherlands) using Ir-192.

At the discretion of the attending physician, weekly CDDP 40 mg/m^2^ was used in 10 patients concurrently with EBRT. In general, patients with bulky disease, good performance status and adequate organ function were selected for the candidate for the administration of concurrent chemoradiation. Patients were seen in follow up 1 week after HDR-ISBT for a skin check, then every 1-2 months for 2 years, every 3-4 months for 5 years, and every 6-12 months thereafter.

When adding doses of EBRT, HDR-ISBT, and HDR-ICBT, we used the equivalent dose in 2 Gy fractions (EQD_2_) according to the LQ model [10,11]. For re-irradiated patients, prior central pelvic EBRT doses were also added to EQD_2_ for OARs. For those who had prior HDR-ICBT without DVH parameters of OARs because of lack of three dimensional dose calculations, it was difficult to estimate EQD_2_ for OARs. Therefore, prescribed dose for tumor in EQD_2_ (α/β = 10) was converted to EQD_2_ for late responding tissue (α/β = 3) and added together. Time interval between prior RT and the current RT was not taken into consideration in this analysis.

Rectum and bladder were contoured as a whole organ. Vaginal wall was extracted with a thickness of 4 mm on all CT images according to the Vienna group [12]. As for rectum and bladder, dosimetric parameter of D_2cc_ was used because these values have been validated by several studies [6-8]. On the other hand, there is no validated parameter for vaginal dose; therefore D_0.5cc,_ D_1cc,_ D_4cc_, D_6cc_, and D_8cc_ were calculated along with D_2cc_ for vaginal wall dose volume parameters.

Late vaginal morbidities were retrospectively evaluated according to LENT-SOMA scales [13]. Because morbidity scores were evaluated retrospectively in this study, we focused on only vaginal ulcer which could be regarded as one of the severest symptoms and could be retrieved accurately from medical records.

Student’s unpaired t-test was used to compare the continuous variables and Pearson’s chi-square test to compare categorical variables. A *p* value of < 0.05 was considered as statistically significant. In addition, calculation of the area under the curve (AUC) of receiver operating characteristics (ROC) was used to determine the most predictive dosimetric parameter of vaginal ulcer. The predictive values of parameters were evaluated based on the AUC. The optimal threshold for each parameter was defined as the point yielding the minimal value for (1 - sensitivity) ^2^ + (1 – specificity) ^2^, which is the point on the ROC curve closest to the upper left-hand corner [14]. The obtained cutoff point was used for dividing patients into two groups and the incidences of vaginal ulcer were calculated by Kaplan-Meier method with the difference evaluated by log-lank test. All statistical analyses were performed using SPSS Statistics version 18.0 (SAS Institute, Tokyo, Japan).

This retrospective study was approved by the institutional review board of the National Cancer Center.

## Results

There were 44 patients who met the eligibility criteria and 36 patients were alive at the time of the analysis (May 2012). The median follow-up length of living patients was 18.3 months (range, 7.6-39.5 months). The pretreatment characteristics of the 44 patients included in this study are summarized in Table 1. Median age was 56 years (range, 25-89 years). HDR-ISBT was applied as the primary therapy in 14 patients (31.8%) and as the salvage therapy in 30 patients (68.2%). Eight patients (18.2%) had previously received pelvic irradiation, in the form of EBRT and/or ICBT. Twenty four patients were treated with Syed-Neblett perineal template, 17 with free-hand with vaginal applicator and three with free-hand without vaginal applicator. Treatment details are summarized in Table 2. Ten patients underwent concurrent chemotherapy. In most cases HDR-ISBT dose per fraction was 6 Gy. Median total EQD_2_ of CTV D_90_ was 67.7 Gy. Median EQD_2_ of D_2cc_ for rectum and bladder was 60.8 Gy and 58.1 Gy, respectively. Median EQD_2_ of D_0.5cc_, D_1cc_, D_2cc_, D_4cc_, D_6cc_, and D_8cc_ for vaginal wall were 210.7 Gy, 167.3 Gy, 131.5 Gy, 111.6 Gy, 100.0 Gy, and 83.2 Gy, respectively. Table 3 shows EQD_2_ of rectum, bladder and vaginal wall for the patients with or without prior pelvic radiation therapy. For re-irradiation patients, median EQD_2_ of D_2cc_ for rectum and bladder, D_0.5cc_, D_1cc_, D_2cc_, D_4cc_, D_6cc_ and D_8cc_ for vaginal wall was 91.1 Gy, 100.9 Gy, 260.3 Gy, 212.3 Gy, 170.1 Gy, 117.1 Gy, 105.2 Gy, and 94.7 Gy, respectively. For those without prior radiation therapy, median EQD_2_ of D_2cc_ for rectum and bladder, D_0.5cc_, D_1cc_, D_2cc_, D_4cc_, D_6cc_ and D_8cc_ for vaginal wall was 56.3 Gy, 54.3 Gy, 147.4 Gy, 126.2 Gy, 108.0 Gy, 103.5 Gy, 94.7 Gy, and 80.7 Gy, respectively (Table 3). In EQD_2_ of D_2cc_ for rectum, bladder and vaginal wall the difference was statistically significant (*p* < 0.001, *p* < 0.001, and *p* = 0.001, respectively).

**Table 1:** Patients characteristics (n = 44)

		**Patients (n)**
Median age (years, range)		56 (25-89)
Primary site	Cervix	24 (54.6%)
	Vagina	12 (27.3%)
	Corpus	5 (11.3%)
	Ovary	2 (4.5%)
	Vulva	1 (2.3%)
Primary therapy		14 (31.8%)
	Cervical cancer	4 (9.1%)
	Vaginal cancer	10 (22.7%)
Salvage therapy		30 (68.2%)
	Post ope regidual tumor	5 (11.4%)
	Post ope recurrent tumor	21 (47.7%)
	Post RT recurrent tumor	4 (9.1%)
Histology	Scc	25 (56.8%)
	Adeno	16 (36.4%)
	Others	3 (6.8%)
Prior pelvic RT^*^	Yes	8 (18.2%)
	No	36 (81.8%)
Median tumor size (cm, range)		3.6 (1.0-8.0)
Pelvic LN^†^ metastais	Yes	11 (25%)
	No	33 (75%)

*RT radiation therapy.

^†^*LN *lymph node.

**Table 2:** Treatment details (n = 44)

	**Median range**
Central pelvic dose of EBRT^*^ (Gy)	30 (0-50)
No. of needles used in HDR-ISBT^†^	15 (5-29)
HDR-ISBT^†^ fractions	4 (3-9)
HDR-ISBT^†^ dose per fraction (Gy)	6 (4-6)
CTV^††^ (ml)	35.1 (2.4-142.1)
CTV^††^ D_90 _in EQD_2_^||^ (Gy)	67.7 (48.8-94.2)
Rectum D_2cc_^¶^ in EQD_2_^||^ (Gy)	60.8 (30.5-114.3)
Bladder D_2cc_^¶^ in EQD_2_^||^ (Gy)	58.1 (7.3-120.3)
Vaginal wall D_0.5cc_^¶^ in EQD_2_^||^ (Gy)	210.7 (51.5-468.1)
Vaginal wall D_1cc_^¶^ in EQD_2_^||^ (Gy)	167.3 (49.9-352.1)
Vaginal wall D_2cc_^¶^ in EQD_2_^||^ (Gy)	131.5 (43.7-294.4)
Vaginal wall D_4cc_^¶^ in EQD_2_^||^ (Gy)	111.6 (34.0-200.8)
Vaginal wall D_6cc_^¶^ in EQD_2_^||^ (Gy)	100.0 (20.4-173.7)
Vaginal wall D_8cc_^¶^ in EQD_2_^||^ (Gy)	83.2 (10.3-144.4)
Concurrent chemotherapy	
Yes	10 patients
No	34 patients

^*^EBRT: external beam radiation therapy.

^†^HDR-ISBT: high-dose-rate interstitial brachytherapy.

^††^CTV: clinical target volume.

^||^EQD2: equivalent dose in 2 Gy fractions.

^¶^D0.5cc, D1cc, D2cc, D4cc, D6cc, D8cc: most exposed 0.5, 1, 2, 4, 6 and 8 cm3 of tissue.

**Table 3:** DVH parameters for bladder and vaginal wall with or withour prior radiation therapy

	**Prior pelvic RT**^**∫ **^**(+) (n = 8)**	**Prior pelvic RT**^**∫ **^**(-) (n = 36)**	***p *** **value**
eMedian rectum D_2cc_^†^ (EQD_2_^*^, Gy, range)	91.1 (71.0-114.3)	56.3 (30.5-82.7)	< 0.001^*^
Median bladder D_2cc_^†^ (EQD2^*^, Gy, range)	100.9 (69.7-120.3)	54.3 (7.3-82.7)	< 0.001^*^
Median vaginal wall D_0.5cc_^†^ (EQD2^*^, Gy, range)	260.3 (59.9-349.3)	147.4 (47.9-267.3)	0.109
Median vaginal wall D_1cc_^†^ (EQD2^*^, Gy, range)	212.3 (58.2-277.5)	126.2(33.6-182.7)	0.013
Median vaginal wall D_2cc_^†^ (EQD2^*^, Gy, range)	170.1 (56.6-247.5)	108.0 (31.7-150.9)	0.001^*^
Median vaginal wall D_4cc_^†^ (EQD2^*^, Gy, range)	117.1 (34.0-200.8)	103.5 (39.1-139.4)	0.139
Median vaginal wall D_6cc_^†^ (EQD2^*^, Gy, range)	105.2 (33.0-173.7)	94.7 (20.4-138.7)	0.097
Median vaginal wall D_8cc_^†^ (EQD2^*^, Gy, range)	94.7 (32.4-144.4)	80.7 (10.3-130.4)	0.105

^∫^RT: radiation therapy.

^*^EQD2: equivalent dose in 2 Gy fractions.

^†^D0.5cc, D1cc, D2cc, D4cc, D6cc, D8cc: most exposed 0.5, 1, 2, 4, 6, and 8 cm3 of tissue.

**Figure 1:**
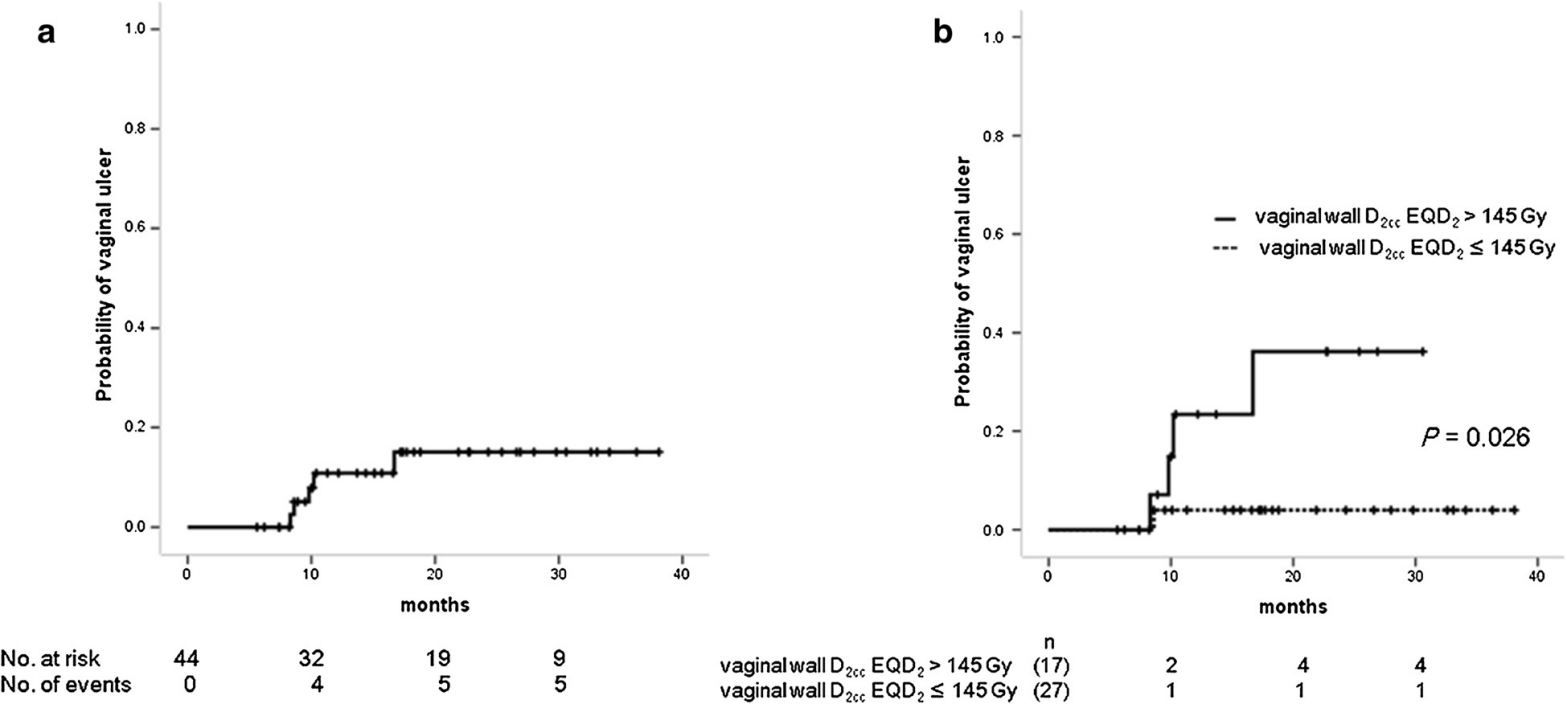
**Cumulative incidene of vaginal ulcer. a**: Cumulative incidence of vaginal ulcer. **b**: Cumulative incidence of vaginal ulcer stratified by vaginal wall D_2cc _145 Gy in EQD_2_.

As for late morbidities of vagina, five patients experienced vaginal ulcer after HDR-ISBT. All of vaginal ulcer occurred within two years after completion of the HDR-ISBT. Patient characteristics and objective/management scores of vaginal ulcer according to LENT-SOMA are summarized in Table 4. Two patients had superficial and > 1 cm^2^ vaginal ulcer and three had vaginal fistula (two vesicovaginal fistulae and one vesicovaginorectal fistula). Three out of the five patients had prior pelvic irradiation and the interval between prior pelvic irradiation and secondary pelvic irradiation was 15, 27, and 40 months, respectively. All of the three patients with vaginal fistula received hyperbaric oxygen therapy without success. Two underwent surgical intervention (one total cystectomy for massive hematuria and one nephrostomy) for their vesicovaginal fistula, while one was followed up conservatively with a persistent vesicovaginal fistula. The other two patients with grade 2 vaginal ulcer were treated conservatively. The overall 2-year actuarial incidence of vaginal ulcer was 11.4%; 37.5% for re-irradiation patients and 5.6% for those without prior radiation therapy (Figure 1a). Comparison of dose-volume parameters of the vaginal wall is shown in Table 5 for the patient with and without vaginal ulcer. It was shown that the incidence of vaginal ulcer in the patients with prior pelvic irradiation was statistically higher than that of the patients without prior pelvic irradiation (*p* = 0.035). It was also shown that the mean EQD_2_ of vaginal wall D_2cc_ of patients with or without vaginal ulcer was statistically different (*p* = 0.025). There was no relationship between administration of concurrent chemotherapy and manifestation of vaginal ulcer (*p* = 0.256), number of needles used in HDR-ISBT (*p* = 0.293) nor bladder D_2cc_ EQD_2_ (*p* = 0.091). The ROC analysis revealed that vaginal wall D_2cc_ was the best dosimetric parameter predicting the incidence of vaginal ulcer and the cutoff value of 145 Gy in vaginal wall D_2cc_ provided the lowest *p* value in log-rank test (Table 6). Figure 1b shows Kaplan-Meyer curve for the incidence of vaginal ulcer stratified by vaginal wall D_2cc_ 145 Gy in EQD_2_. The 2-year incidence rates of vaginal ulcer in the patients with vaginal wall D_2cc_ equal to or less than 145 Gy in EQD_2_ and over 145 Gy were 3.7% and 23.5%, respectively, with a statistically significant difference (*p* = 0.026).

**Table 4:** Patient characteristics who developed vaginal ulcer

**Patient no.**	Age at HDR-ISBT^*^	**Primary site**	**Prior pelvic RT**	Interval between prior RT and HDR-ISBT^* ^**(mo)**	HDR-ISBT^* ^**with/without EBRT**^**††**^	**Total vaginal wall D**_**0.5cc**_^**#**^**/D**_**1cc**_^**#**^**/D**_**2cc**_^**# **^**in EQD**_**2**_^**## **^**(Gy)**	**LENT SOMA**^**¶ **^**objective score**	**LENT SOMA**^**¶ **^**management score**
1	40	Cervix	WPRT^†^ 45 Gy/25fr + EBRT^††^ boost 15 Gy/5fr	27	HDR-ISBT^*^ 36 Gy/9fr	272.1/202.6/169.1	4	4
2	51	Cervix	None	None	WPRT^†^ 30 Gy/15fr + CS^||^ 20 Gy/10fr + HDR-ISBT^*^ 24 Gy/4fr	215.2/171.8/145.4	4	3
3	64	Corpus	None	None	WPRT^†^ 30 Gy/15fr + HDR-ISBT^*^ 30 Gy/5fr	196.6/141.5/109.1	2	1
4	64	Cervix	WPRT^†^ 40 Gy/20fr + CS 10 Gy/5 + HDR-ICBT 18 Gy/3fr	40	HDR-ISBT^*^ 48 Gy/8fr	465.4/352.1/294.4	2	1
5	67	Cervix	WPRT^†^ 50 Gy/50fr + HDR-ICBT 12 Gy/3fr	15	HDR-ISBT^*^ 42 Gy/7fr	234.0/211.1/193.5	4	3

^*^HDR-ISBT: high-dose-rate interstitial brachytherapy.

^†^WPRT: whole pelvis radiation therapy.

^††^EBRT: external beam radiation therapy.

^||^CS: radiation therapy with center shielding.

^¶^LENT-SOMA: Late Effects of Normal Tissues - Subjective, Objective, Management, Analytic.

^#^D0.5cc, D1cc, D2cc: most exposed 0.5, 1 and 2 cm3 of tissue.

^##^EQD2: equivalent dose in 2 Gy fractions.

**Table 5:** Clinical predictors of vaginal ulcer

**Characteristic**	**Vaginal ulcer (+) (n = 5)**	**Vaginal ulcer (-) (n = 39)**	**p value**
Prior pelvic RT^*^	3	5	0.035^*^
Yes	2	34	
No			
Concurrent chemotherapy			0.256
Yes	0	10	
No	5	29	
Median number of needles used in HDR-ISBT^∫^ (range)	14 (10-24)	15 (5-29)	0.293
Median CTV^†^ (ml, range)	54.7 (17.7-114.0)	34.7 (2.4-142.1)	0.271
Median rectum D_2cc_^††^ (EQD_2_^||^, Gy, range)	84.2 (34.0-100.7)	57.9 (30.5-114.3)	0.118
Median bladder D_2cc_^††^ (EQD_2_^||^, Gy, range)	69.3 (37.4-113.5)	57.7 (7.3-120.3)	0.091
Median vaginal wall D_0.5cc_^††^ (EQD_2_^||^, Gy, range)	206.4 (106.6-349.3)	149.4 (47.9-310.1)	0.243
Median vaginal wall D_1cc_^††^ (EQD_2_^||^, Gy, range)	169.1 (91.6-277.5)	127.9 (33.6-220.8)	0.096
Median vaginal wall D_2cc_^††^ (EQD_2_^||^, Gy, range)	152.5 (71.1-247.5)	109.0 (31.7-201.9)	0.025^*^
Median vaginal wall D_4cc_^††^ (EQD_2_^||^, Gy, range)	115.5 (83.8-200.8)	110.6 (34.0-153.2)	0.152
Median vaginal wall D_6cc_^††^ (EQD_2_^||^, Gy, range)	102.5 (60.4-173.7)	99.5 (20.4-146.3)	0.266
Median vaginal wall D_8cc_^††^ (EQD_2_^||^, Gy, range)	82.0 (47.6-144.4)	84.3 (10.3-140.3)	0.511

^*^RT: radiation therapy.

^∫^HDR-ISBT.

^†^CTV: clinical target volume.

^||^EQD2: equivalent dose in 2 Gy fractions.

^††^D0.5cc, D1cc, D2cc, D4cc, D6cc, D8cc: most exposed 0.5, 1, 2, 4, 6, and 8 cm3 of tissue.

**Table 6:** Dosimetric predictors for the development of vaginal ulcer

			**2-y incidence ofvaginal ulcer (%)**	
**Parameter**	**ROC**^**† **^AUC^*^	**Cutoff**^**¶**^		**P value**^**#**^
Vaginal wall D_0.5cc_^||^ (EQD_2_^††^)	0.667	≤195 Gy	0.0	0.058
		>195 Gy	18.5	
Vaginal wall D_1cc_^||^ (EQD_2_^††^)	0.682	≤171 Gy	4.2	0.091
		>171 Gy	20.0	
Vaginal wall D_2cc_^||^ (EQD2^††^)	0.733	≤145 Gy	3.7	0.026^*^
		>145 Gy	23.5	
Vaginal wall D_4cc_^||^ (EQD_2_^††^)	0.618	≤83 Gy	0.0	0.119
		>83 Gy	15.6	
Vaginal wall D_6cc_^||^ (EQD_2_^††^)	0.569	≤86 Gy	5.6	0.323
		>86 Gy	15.4	
Vaginal wall D_8cc_^||^ (EQD_2_^††^)	0.559	≤75 Gy	5.6	0.323
		>75 Gy	15.4	

^*^AUC: area under the curve.

^†^ROC: receiver operator characteristic.

^††^EQD2: equivalent dose in 2 Gy fractions.

^||^D0.5cc, D1cc, D2cc, D4cc, D6cc, D8cc: most exposed 0.5, 1, 2, 4, 6, and 8 cm3 of tissue.

^¶^Cutoff refers to the most predictive value from the AUC of ROC curve.

^#^ Univariate analysis by log-rank test.

## Discussion

Although the Manchester method of ICBT for the cervical cancer was developed to avoid the occurrence of radiation induced vaginal ulcer and necrosis, vaginal ulcer is now very rarely encountered because vaginal wall is relatively radioresistant and typical ICBT delivers radiation dose less than the tolerance of the relatively radioresistant vaginal wall. In a retrospective study of cervical cancer patients using EBRT and the film based low-dose rate (LDR) brachytherapy, Samuel et al. showed that vaginal tolerance dose was above 150 Gy [15]. In recent advancement of image guided brachytherapy (IGBT), rectum and bladder doses were recommended to be reported in the treatment of ICBT for cervical cancer but vagina was not mentioned as OAR [9]. In the GEC-ESTRO working group (II) or American Brachytherapy Society guidelines, vagina was taken into consideration for OAR but it was stated that the vaginal dose volume parameters still need to be defined [16,17]. Dimopoulos et al. reported clinical result of primary vaginal cancer treated with IGBT and they experienced two vaginal fistulae and one periurethral necrosis. However they did not specify DVH parameters of vaginal wall with vaginal complication [18]. Lee et al. reported in detail the toxicity analysis of CT based HDR-ISBT for gynecologic malignancies. They reported that D_2cc_ for the rectum was a reliable predictor of late rectal complication; however because of limited number of events it was not able to explore the DHV parameters for vaginal complication [5]. Recently, Vienna group tried to find out DVH parameters that correlate with vaginal late morbidities but vaginal D_2cc_ did not relate with the vaginal morbidities [12]. The calculation method was the same as the current study, which was composed of EBRT and ICBT/ISBT and normalized to 2 Gy per fraction (EQD_2_) using the linear-quadratic model with α/β of 3 Gy for the vaginal morbidities [10-12,16]. The difference between Vienna group and the current study was that there were more patients with severe vaginal morbidities in the current study, presumably because there were more patients who received re-irradiation and current study excluded the patients treated with HDR-ICBT. HDR-ISBT delivers higher dose to the vaginal wall than HDR-ICBT because the multiple needle applicators directly contact vaginal wall. According to the current results, after vaginal wall D_0.5cc,_ D_1cc,_ D_2cc,_ D_4cc,_ D_6cc,_ and D_8cc_ having been compared, vaginal wall D_2cc_ was found to be the most relevant DVH parameter predicting the incidence of vaginal ulcer. ROC analysis also showed that vaginal wall D_2cc_ of 145 Gy in EQD_2_ can be used as clinical cutoff dose predicting vaginal ulcer. This figure is quite similar to the vaginal tolerance dose of 150 Gy derived from a retrospective study of LDR brachytherapy which was previously mentioned [15]. The current report is the first one concerning about vaginal DVH parameter and complication using modern era of three-dimensional image-guided brachytherapy. It was also found in this study that the history of prior pelvic irradiation was another significant predictive factor for vaginal ulcer (Table 5). Lee et al. reported a patient with colovaginal fistula with previous EBRT [5,7]. As shown in Table 3, both rectum and bladder D_2cc_ was significantly higher in patients with prior pelvic irradiation than those without prior pelvic irradiation. However both rectum and bladder D_2cc_ was not in itself a significant prognostic factor for vaginal ulcer and could not be used as a surrogate indicator (Table 5).

There were several limitations in this study. Contouring of the vagina was not based on MRI but CT, which is inferior to MRI in tissue contrast. However because 41 out of 44 patients were inserted either cylinder or mold into their vagina, contouring of vagina was considered to be precise. The time interval between the prior pelvic RT and HDR-ISBT was not taken into consideration for the calculation of the total dose for OARs. Additionally, this study was a retrospective study with small number of patients with heterogeneous tumor origin, heterogeneous treatment applied, small number of events, and with short follow-up period. Therefore we should be cautious about the results of the current study. However even tumor origin differed greatly in current cohorts of study, it is considered to be feasible because the main concern in current study was focused on only the vaginal toxicity.

It should be stressed that with the introduction of HDR-ISBT in gynecological malignancies and increment of vaginal dose, vaginal tolerance dose must be taken into consideration. Further discussion and validation of vaginal DVH parameters in image-guided brachytherapy in a multicenter prospective study is needed.

## Conclusions

The DVH parameters for vagina are essential for treatment planning and optimization in image based HDR-ISBT in gynecological malignancies. Vaginal wall D_2cc_ in EQD_2_ should be monitored and be kept under 145 Gy in order to avoid vaginal ulcer. Also in patients with prior pelvic irradiation, vaginal wall dose including the prior radiation dose should be kept lower than 145 Gy.

## Consent

Written informed consent was obtained from the patient for the publication of this report and any accompanying images.

## Abbreviations

HDR-ISBT: High-dose rate interstitial brachytherapy; EQD2: Dose in equivalent in 2 Gy fractions; ICBT: Intracavitary brachytherapy; ISBT: Interstitial brachytherapy; DVH: Dose volume histogram; EBRT: External beam radiation therapy; GTV: Gross tumor volume; CTV: Clinical target volume; OAR: Organ at risk; AUC: Area under the curve; ROC: Receiver operating characteristics; IGBT: Image guided brachytherapy; PDR: Pulsed dose rate.

## Competing interests

The authors declare that they have no competing interests.

## Authors’ contributions

TK, MS, RY, KH, MK, SS, KT, KY, KI, MM, and YI performed the treatment. NM and JI analyzed the data and wrote the manuscript. All authors read and approved the final manuscript.
